# Inhibition of the Nav1.7 Channel in the Trigeminal Ganglion Relieves Pulpitis Inflammatory Pain

**DOI:** 10.3389/fphar.2021.759730

**Published:** 2021-12-08

**Authors:** Minjee Kwon, Il Young Jung, Myeounghoon Cha, Bae Hwan Lee

**Affiliations:** ^1^ Department of Nursing, Kyungil University, Gyeongsan, South Korea; ^2^ Department of Physiology, Yonsei University College of Medicine, Seoul, South Korea; ^3^ Department of Conservative Dentistry and Oral Science Research Center, Yonsei University College of Dentistry, Seoul, South Korea; ^4^ Brain Korea 21 PLUS Project for Medical Science, Yonsei University College of Medicine, Seoul, South Korea

**Keywords:** Nav1.7 channel, pulpitis, pain, endodontics, trigeminal ganglion

## Abstract

Pulpitis causes significant changes in the peripheral nervous system, which induce hyperalgesia. However, the relationship between neuronal activity and Nav1.7 expression following pulpal noxious pain has not yet been investigated in the trigeminal ganglion (TG). The aim of our study was to verify whether experimentally induced pulpitis activates the expression of Nav1.7 peripherally and the neuronal activities of the TGs can be affected by Nav1.7 channel inhibition. Acute pulpitis was induced through allyl isothiocyanate (AITC) application to the rat maxillary molar tooth pulp. Three days after AITC application, abnormal pain behaviors were recorded, and the rats were euthanized to allow for immunohistochemical, optical imaging, and western blot analyses of the Nav1.7 expression in the TG. A significant increase in AITC-induced pain-like behaviors and histological evidence of pulpitis were observed. In addition, histological and western blot data showed that Nav1.7 expressions in the TGs were significantly higher in the AITC group than in the naive and saline group rats. Optical imaging showed that the AITC group showed higher neuronal activity after electrical stimulation of the TGs. Additionally, treatment of ProTxII, selective Nav1.7 blocker, on to the TGs in the AITC group effectively suppressed the hyperpolarized activity after electrical stimulation. These findings indicate that the inhibition of the Nav1.7 channel could modulate nociceptive signal processing in the TG following pulp inflammation.

## Introduction

Pain is an intricate phenomenon, and orofacial pain, which is a multifactorial experience, includes distressing sensory response and emotional modality (Estrela et al., 2011; Schuh et al., 2019). Dental pain associated with pulp inflammation, known as pulpitis, is quite common, and ectopic persistent pain and hyperalgesia are frequently observed after pulpitis (Matsuura et al., 2013; Alelyani et al., 2020; Sooratgar et al., 2021). Although there have been many studies regarding the identification of pulpitis (Hargreaves, 2011; Rechenberg et al., 2016; Filippini et al., 2018), the fundamental mechanisms of pain initiation and maintenance in pulpitis are still not fully understood; however, they are associated with sodium channels in the trigeminal nerves. There are many origins of pain in the orofacial region; among them, the trigeminal nerve fiber, which is the primary nerve fiber, predominantly innervates the orofacial region. Pain sensation from the orofacial region is transmitted to the TG, which is composed of three main branches: the ophthalmic (V1), maxillary (V2), and mandibular (V3) nerve branches. Pulpitis is generally a consequence of injury to one or more branches (nearly V2 and/or V3) (Maarbjerg et al., 2017). Alterations in primary afferent nerve fibers result in increased excitability, which causes hyperalgesia (Byers and Närhi, 1999). These functional changes may be manipulated by specific signaling components in neurons (Sun et al., 2019). Ectopic neuronal activity triggered by increasing sensitization is coincident with changes in the expressions of several ion channels and receptors (Julius and Basbaum, 2001; Davies et al., 2006).

Voltage-gated sodium channels (VGSCs) are transmembrane protein complexes that play critical roles in the formation and conduction of action potentials in response to stimuli exceeding threshold values in neuronal cells (Hameed, 2019). Many studies have shown that VGSCs control nerve excitability, inclusive of action potentials (Padilla et al., 2007; Luo et al., 2010). Moreover, they play an essential role in the conduction of electrical excitability (Rogers et al., 2006; Bi et al., 2016; Liu et al., 2019; Sun et al., 2019). Previous studies have indicated that sensory neurons alter the expression of VGSCs after inflammatory lesions (Wood et al., 2004; Dib-Hajj et al., 2010). The respective isoforms exhibit distinct distributions in the nervous system and have a noticeable electrophysiological composition (Cummins et al., 2007; Luo et al., 2010). In particular, Nav1.7 is highly enriched in the dorsal root ganglion (DRG), TG, sympathetic ganglia, and nociceptors of the peripheral nervous system (Toledo-Aral et al., 1997; Luiz and Wood, 2016; Liu et al., 2019; McDermott et al., 2019). Various pain syndromes like erythromelalgia and paroxysmal extreme pain disorder are caused by aberrant Nav1.7 (Cummins et al., 2004; Choi et al., 2011). Thus, Nav1.7 performs a noticeable function in pain perception, particularly inflammatory pain (Emery et al., 2016; Bi et al., 2017). Previous studies have found that nociceptor-specific knockout of Nav1.7 abolishes inflammation-induced hyperalgesia (Julius and Basbaum, 2001; Yeomans et al., 2005; Zhang and Gan, 2017). Indeed, studies have demonstrated that a lack of Nav1.7 in sensory neurons leads to diminished hypersensitivity in inflammatory pain models (Minett et al., 2012; McDermott et al., 2019).

Considering that Nav1.7 is related to inflammatory pain, we hypothesized that inhibition of Nav1.7 in the TG could relieve the hyperalgesia in pulpitis. The present study aimed to investigate the mechanisms underlying allyl isothiocyanate (AITC)-induced pulpitis and Nav1.7 expression changes in the TG. Furthermore, this study could suggest the possible clinical treatments for severe painful dental diseases.

## Materials and Methods

### Animals

Male Sprague-Dawley rats (200–220 g, Koatech, Pyeongtaek, Korea) were subjected to all experiments in the Laboratory Animal Facility at Yonsei University, Seoul, Republic of Korea; these experiments were approved by the Institutional Animal Care and Use Committee (2019-0093). The animals were housed in temperature- (22 ± 2°C) and humidity-controlled rooms (50 ± 10%) within plastic cages containing soft bedding in a 12-h light/dark cycle with food and water provided *ad libitum*. Rats were randomly assigned to one of the following three groups: the no pulp inflammation group (naive group; *n* = 12), the pulp exposure with saline application group (saline group; *n* = 12), and the pulp exposure with allyl isothiocyanate (AITC) application group (AITC group; *n* = 12). All efforts were made to minimize animal suffering and reduce the number of animals used.

### Inflammation of Tooth Pulp

The surgical procedure was performed as previously described (Cha et al., 2020). The rats were anesthetized using intraperitoneal (i.p.) sodium pentobarbital (50 mg/kg) before being placed in the supine position. The rat’s mouth was gently opened to expose the pulp of the left maxillary first molar (M1) using a low-speed dental drill with a 2 mm round tungsten carbide bur. For rats in the AITC group, a small piece of dental paper point (diameter, 0.15 mm; length, 20 mm) soaked in AITC (Sigma-Aldrich, Milwaukee, WI, US) was applied for 2 min to the exposed M1 tooth pulp. In the saline group rats, the exposed pulp was treated similarly, while saline was applied instead of AITC. The exposed pulp cavity was then sealed with visible light-cured composite resin (SDR®, Dentsply Sirona, York, PA, US) using Singlebond Universal (3M ESPE, Maplewood, MI, US).

### Behavioral Testing

On day 3 after AITC or saline administration to M1, behavioral tests were conducted to evaluate the intensity of the pulpitis-induced pain. Rats were habituated for 10 min to the test chambers, which consisted of a metal mesh floor under individual Plexiglas boxes (9 × 9 × 18 cm) on an elevated table. In this study, we aimed to evaluate the degree of pain by measuring the abnormal response of pain induced by pulpitis using four behavior measurement methods (Cha et al., 2020). The spontaneous face-grooming activity was monitored and videotaped for 10 min. Different types of behaviors were assessed, including face-wash strokes, paw-licks, ear grasps, and chin rubs. All face-grooming actions were measured as separate events. For each observation session, the total duration of face-grooming events was evaluated. Videotaped behaviors were analyzed offline by assessors who were blinded to the group information of the rats.

### Tissue Collection and Preparation

For immunohistochemistry (IHC), after the behavioral testing, the rats were perfused using 0.9% NaCl and 4% paraformaldehyde in sodium phosphate buffer, and the maxillary bone and first molar were removed and post-fixed in the same fixative solution for 24 h and subjected to decalcification in 17% ethylenediaminetetraacetic acid (EDTA, pH 7.0 with addition of NaOH) for 3 weeks. Additionally, the extracted ipsilateral TG specimens were collected and post-fixed overnight at 4°C before cryoprotection in 30% sucrose in phosphate-buffered saline (PBS, pH 7.4) for 24 h.

### Immunohistochemistry

To verify pulpal inflammation, tooth specimens were cryosectioned at 12-μm intervals and stained with hematoxylin and eosin. Neutrophil infiltration was used as a criterion for pulpal inflammation. To identify the changes in TGs due to AITC-induced pain, the expression levels of Nav1.7 were assessed in the collected brainstems and ipsilateral TG specimens. Longitudinal sections of the TG specimens were obtained using a cryostat (Microm HM 525; Thermo Scientific, Walldorf, Germany). The collected tissues were blocked using 5% normal goat serum containing 0.1% Triton X-100 for 1 h and then reacted with rabbit anti-Nav1.7 anti-serum (1:500; Cell Signaling Technology, Danvers, MA, United States). The sections were incubated for 2 h at 22 ± 2°C with secondary antibodies consisting of biotinylated goat anti-rabbit IgG serum (1:200) and then washed with PBS and further incubated for 2 h with Alexa Fluor 488 and Cytm 3-conjugated AffiniPure F (ab’)2 Fragment Donkey Anti-Mouse IgG secondary antibodies (1:1,000; Jackson ImmunoResearch, West Grove, PA, United States). DAPI was used for counterstaining. Immunofluorescence images were obtained using an LSM700 confocal microscope (Zeiss, Oberkochen, Germany). Using the ZEN program (Zen 2.3 black software, Carl Zeiss), we set the reference threshold value for non-positive immunoreactive response, and the neurons selected above the threshold value were countered. The number of cells with colocalized expression of Nav1.7 was quantified. Three image stacks per rat (*n* = 4 rats/group) were used for image analysis, and standardized regions of interest were outlined in the TG sections to encompass neuronal cells of the maxillary branch (V2) of the trigeminal nerve at the intersection with the mandibular branch (V3) of the trigeminal nerve.

### Western Blot Analysis

On three days post-injection, targeted TGs were collected, immediately frozen in liquid nitrogen, and stored at −70°C. TG explants were homogenized in lysis buffer (PRO-PREP; Intron Biotechnology, Pyeongtaek, Korea) containing phosphatase inhibitors (PhosSTOP; Roche, Mannheim, Germany). The supernatant was collected, and samples with the same amounts of protein were separated and transferred to a membrane (Merck Millipore, Darmstadt, Germany). The membrane was incubated with 5% bovine serum albumin solution for 1 h at room temperature and then incubated with anti-Nav1.7 antibody (ab65167, 1:1,000; Abcam, Cambridge, United Kingdom) and β-actin antibody (no.4970, 1:10,000; Cell Signaling Technology) overnight at 4°C. The membrane was then incubated with anti-rabbit horseradish peroxidase-conjugated secondary antibody (No. 7074, 1:10,000; Cell Signaling Technology) for 2 h at 20°C. Proteins were visualized by applying a chemiluminescent substrate (GE Healthcare, Little Chalfont, United Kingdom) and observed using the LAS system (LAS 4000; GE Healthcare). β-Actin was used as a loading control.

### Optical Imaging With Voltage-Sensitive Dye for Neuronal Activity Recording in TGs

Three days post-injection, the rats were anesthetized using urethane (50 mg/kg, i.p.). The dissected TGs were isolated in a optical camber. The chamber was filled with artificial cerebrospinal fluid (aCSF) equilibrated with a gas mixture (5% CO_2_ in O_2_; pH 7.4). The composition of the mock CSF was as follows (in mM): NaCl, 126 mM; KCl, 5 mM; CaCl_2,_ 2 mM; MgSO_4_, 2 mM; NaH_2_PO_4_, 1.2 mM; NaHCO_3_, 26 mM; and glucose, 30 mM. TGs were quickly isolated and prepared for optical imaging in the recording chamber. In brief, for staining, preparations were kept for 1 h in aCSF containing a voltage-sensitive dye (VSD) (Di-2-ANEPEQ, 50 mg/ml in saline, Molecular Probes, Eugene, OR, US). After staining, the staining dye was exchanged with fresh aCSF solution. After 10 min of washing, optical imaging and data analysis were performed using a MiCAM02 hardware and software package (BrainVision, Tokyo, Japan). For optical imaging, we used a fixed-stage upright fluorescence microscope (BX51WI, Olympus, Tokyo, Japan) and a high-resolution MiCAM02 camera.

To record the activation of TGs, we used a halogen lamp (150 W). Light from the filtered tungsten-halogen lamp (480–550 nm) was reflected onto the surface of the TGs via a dichroic mirror. Fluorescence images were acquired using an absorption filter at a rate of 3.7 ms/frame (MiCAM02, BrainVision). The charge-coupled device-based camera captured a 4 × 3 mm^2^ imaging area, consisting of 184 × 124 pixels. A concentric bipolar electrode was used for electrical stimulation of the TG. Twenty consecutive images in response to the electrical stimulation of V2 (200 ms delay, 1 ms pulse width, 3 s interstimulus intervals) were averaged to reduce background noise and artifacts. Stimulation was performed with two intensities of 1 and 3 mA. In order to observe the normal nociceptive responses, 1 mA applied in TG. And the hyperactive neuronal responses observed at 3 mA. Image acquisition was triggered by an electrical stimulus. In oreder to verify the effects of Nav1.7 inhibition, ProTxII (selective Nav1.7 blocker, 1 μM, Tocris Bioscience, Bristol, United Kingdom) treated on TGs for 30 min.

### Statistical Analysis

The results of the behavioral tests and immunohistochemical parameters for each experimental group are expressed as the mean and standard error of the mean. An analysis of variance with a Student’s t-test for post-hoc analysis was used to compare the differences in behavioral tests and immunohistochemical labeling (Nav1.7) in the TGs among the groups. The Kruskal-Wallis test with Dunnett’s test for post-hoc analysis was used to compare immunohistochemical labeling (Nav1.7) in the brainstem among the groups. Statistical analyses were performed using SPSS software (version 23.0; IBM Corporation, Armonk, NY, United States). Statistical significance was set at *p* ≤ 0.05. We excluded animals from the analysis when they showed any abnormality (poor general condition, loss of resin, or broken tooth during pulp inflammation) before or during the experiments.

## Results

### Pulp Inflammation and Morphological Changes

**FIGURE 1:**
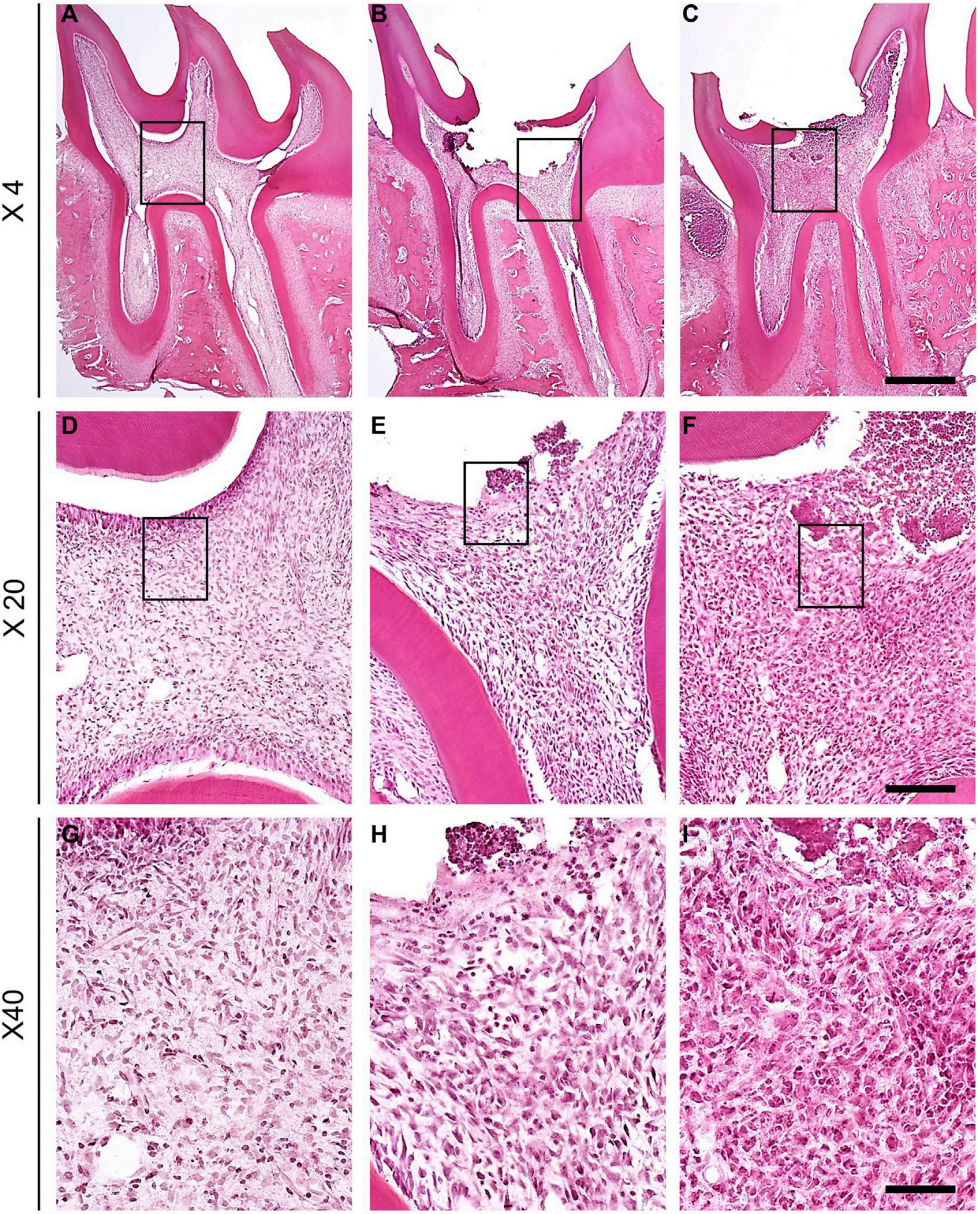
Comparison of cross-section of naïve, saline-treated and molar exposed to AITC. Naïve **(A)**, saline-injected **(B)**, and AITC-injected **(C)** pulp tissues were compared (Scale bar = 200 μm). H&E-stained sections of naïve, saline-treated, and inflamed dental pulp tissue verified the progression of inflammation. **(D–F)** Figures present enlarged black squares in **(A–C)** (Scale bar = 50 μm). **(G–I)** Figures indicate enlarged black squares in **(D–F)** (Scale bar = 10 μm). Figures indicate the AITC-induced pulpal neutrophil changes.

To determine whether dental pulp exposure with AITC injection led to pulpitis, hematoxylin and eosin staining was performed using a section of the first molar on the left maxillary bone. In the comparison of cross-sections of naïve (Figures 1A,D,G), saline-injected (Figures 1B,E,H), and AITC-exposed molars (Figures 1C,F,I), histological signs of pulpitis were verified in the root pulp or periodontal tissue around the apex of AITC-treated group. This suggests that pulp inflammation can affect the radicular portion of the pulpal tissue.

### Assessment of Pain-Like Behaviors After Pulpitis

**FIGURE 2:**
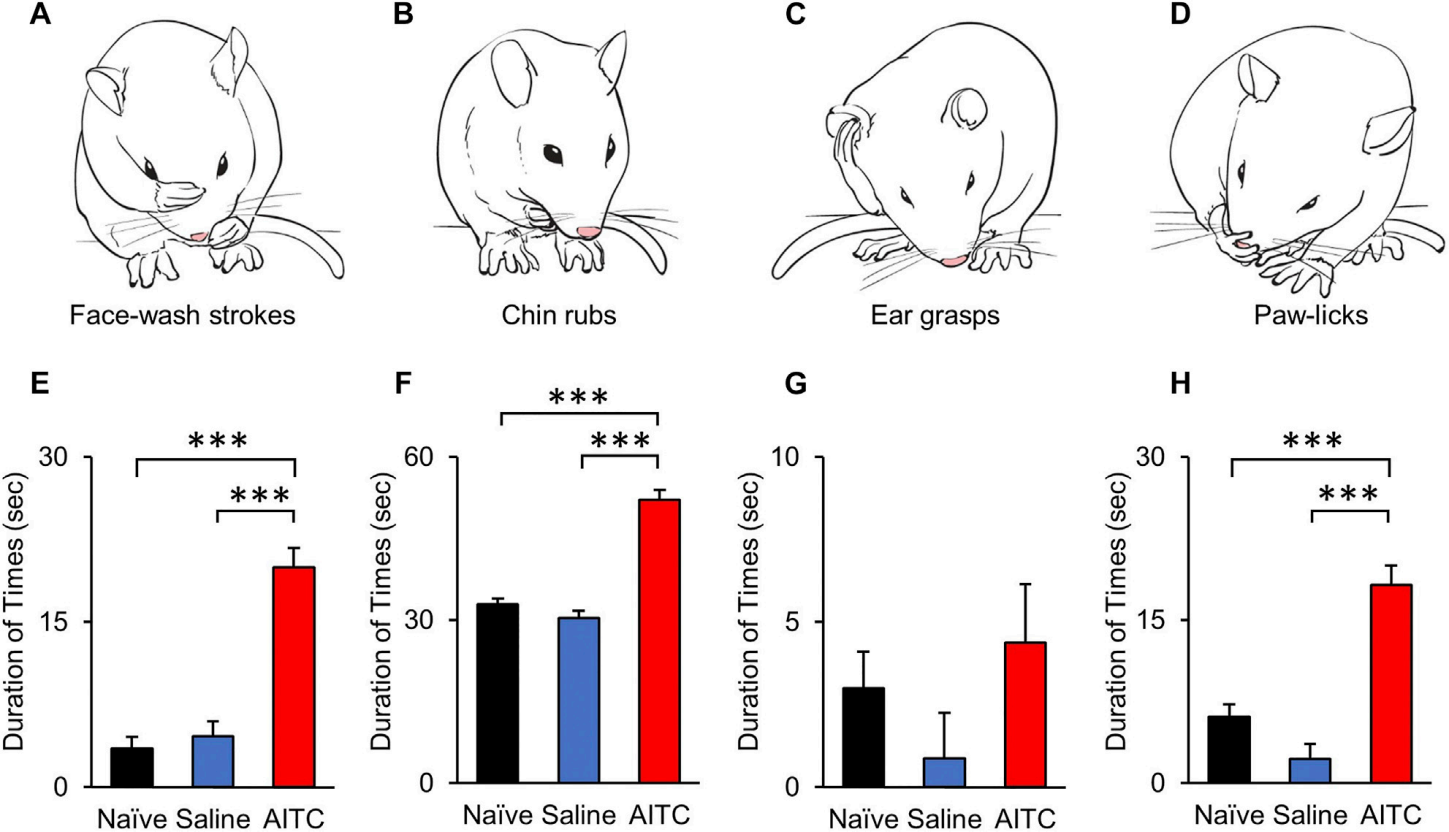
Behavioral analysis after AITC-induced pulpitis. **(A–D)** AITC-induced face grooming behaviors (face-washing, chin-rubs, ear grasps, and paw-licks) are illustrated and compared with naïve and saline-treated groups. Each graph show the duration time of grooming behaviors, face-washing **(E)**, chin-rubs **(F)**, ear grasps **(G)**, and paw-licks **(H)**. Histograms indicate the comparison of naïve, saline-, and AITC-treated rats behaviors. Significant increases were observed in the durations of face-washing, chin rubbing, and paw licking in AITC-treated rats compared with naïve and saline-treated rats (Error bars represent SEM; ***p < 0.001, *n* = 12 each).

To analyze the pain behavioral changes after AITC injection, face-grooming behaviors were compared among naïve, saline, and AITC-injected rats. Characteristic behaviors were analyzed by dividing them into face-washing, chin-rubbing, ear grasps, and paw-licking (Figures 2A–D) (Cha et al., 2020). Three days after the induction of AITC-induced pulpitis, increased facial grooming behaviors were noted in the AITC group rats compared with those in the naive and saline groups. In particular, a significantly longer duration of face-wash strokes was observed in the AITC group (20 ± 1.77 s) than in the naive (3.51 ± 1.10 s) and saline-treated (4.62 ± 1.37 s) groups (Figure 2E). The durations of chin-rubbing (naive 32.87 ± 4.09 s; saline 30.37 ± 9.54 s; AITC 52.12 ± 8.41 s) and paw-licking (naive 6.12 ± 2.23 s; saline 2.25 ± 0.99 s; AITC 18.25 ± 3.32 s) were also significantly longer in the AITC group than in the naive and saline groups, thereby demonstrating a greater nociceptive response in rats treated with AITC (Figures 2F,H). The mean duration of ear grasping was longer than that in the naive (3.01 ± 1.53 s) and saline groups (0.87 ± 0.39), albeit not significantly (Figure 2G). These results suggest that AITC-induced pulpitis causes orofacial pain and increased grooming behaviors.

### Expression of Nav1.7 in a Rat Model of Pulp Inflammation

**FIGURE 3:**
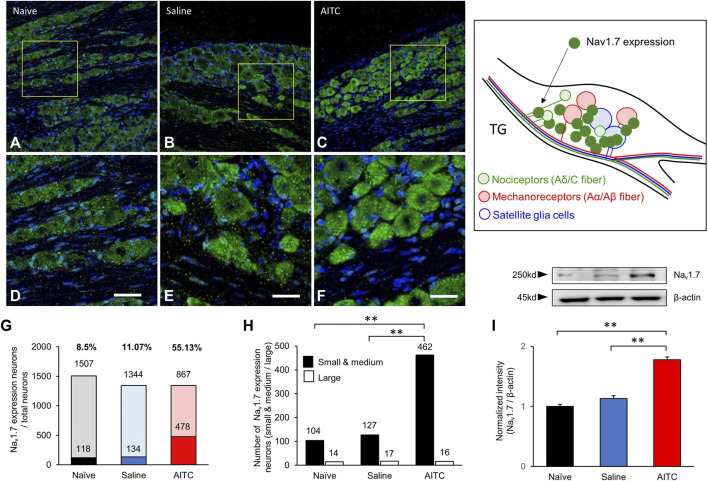
Expressions of Nav1.7 in the TG. **(A–C)** Immunostaining showing Nav1.7 expression in the TG. The protein expression of Nav1.7 increased in the TG in AITC-treated group. **(D–F)** Figures present enlarged yellow squares in a, b, and c, respectively. **(G,H)** The expression ratios were presented and the size-dependent Nav1.7 expression neurons were counted. The highest level of nav1.7 expression was observed in AITC-treated groups. **(I)** Expressions of Nav1.7 were analyzed using western blot (Scale bar = 20 µm, Error bars represent SEM; **p < 0.01, *n* = 4 each).

IHC was used to determine the cellular localization of Nav1.7 in rat TGs on day 3 after treatment. Representative IHC images of TGs from experimental groups showed that Nav1.7 was increased in the AITC-injected TGs compared to those in the naive and saline groups of TGs (Figures 3A–C). To analyze the expression of Nav1.7, 12 different images in each group were randomly selected, and Nav1.7-expressing neurons were counted. In the naive TGs, from a total of 1,507 neurons, only 118 showed Nav1.7 expressions (8.02 ± 1.25%). In addition, in the saline group, only 134 out of 1,344 cells expressed Nav1.7 (10.8 ± 1.24%). However, in the AITC group, 478 neurons out of 1,344 expressed Nav1.7 (35.23 ± 4.61%). TG neurons were analyzed by size in our study, the results showed that Nav1.7 expressions were particularly concentrated in small- and medium-diameter (∼45 μm) TG neurons (Small and medium/large size: Naïve, 104/14; Saline, 127/17; AITC, 462/16). These results showed a significant difference compared to the TGs of the naive and saline groups (Figures 3G,H). Additionally, to determine whether AITC-induced inflammation quantitatively increases Nav1.7 expressions, we analyzed the expression of Nav1.7 in TGs at the end of the experiment. Western blot analysis showed significantly increased Nav1.7 expressions in the AITC group (naive 1 ± 0.03; saline 1.13 ± 0.04; AITC 1.77 ± 0.04). These results suggest that the expressions of AITC-induced Nav1.7 could be observed in TGs.

### Comparison of Neuronal Responses in TGs

**FIGURE 4:**
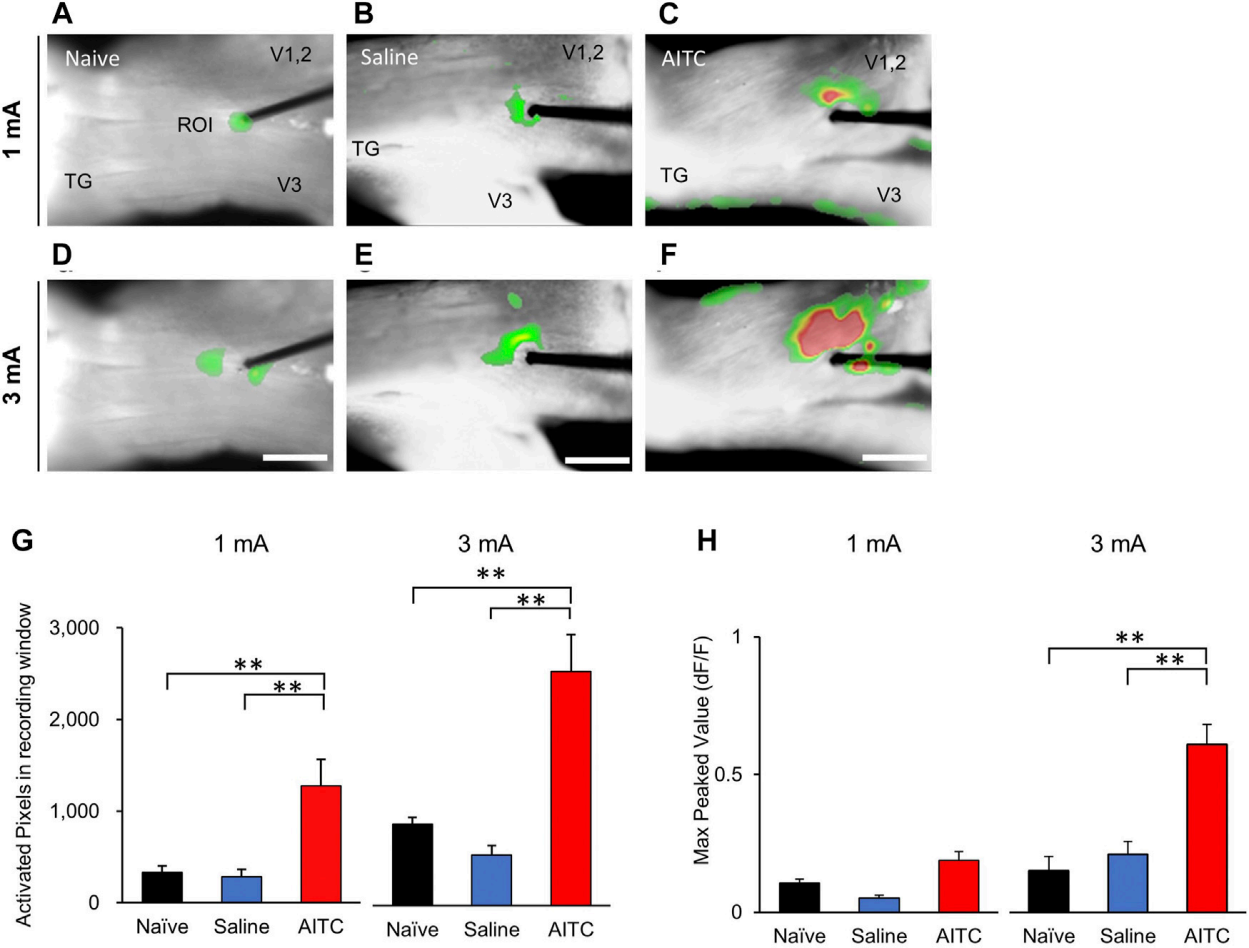
Pulpitis-induced hyperactivities in TG. Comparison of optical signals at 1 **(A–C)** and 3 mA **(D–F)** stimulation in the naïve **(A,D)**, saline-treated **(B,E)**, and AITC-treated **(C,F)** TGs. In the AITC-treated TGs, stimulus-dependent enlarged optical signals were observed. **(G)** Activated pixels were analyzed in different intensity of stimulation. **(H)** Max peaked values after stimulation were analyzed. Compared to naive and sham, more activated pixcels and higher peak activity were observed in AITC-treated group (Error bars represent SEM; **p < 0.01).

In this study, we used VSD imaging to record membrane potential changes in rat TGs. To observe neuronal activity responding to electrical stimulation, we stimulated the V2 region of TGs and recorded the resultant TG neuronal activity. This allowed us to examine the spatial and temporal properties of TG responses to electrical stimulation. In the naive and saline-treated groups, VSD imaging revealed an electrical stimulus-evoked responses in the TGs by 1 mA stimulation (Figures 4A,B). In addition, we found pronounced neuronal activity in the AITC group (Figure 4C). Furthermore, under 3 mA stimulation, images showed higher activity patterns in the AITC group (Figures 4D–F). In comparison with 1 and 3 mA, statistically significant increases in activation were observed at 3 mA. The color-changed pixels of the different images were counted and compared (Figure 4G). In the comparison of activated pixel changes, 1 and 3 mA stimulation in the AITC group of the TGs showed significantly increased neuronal activity compared to the naive and saline groups (naive 1 mA: 329.66 ± 73.93, saline 1 mA: 285.33 ± 78.81, AITC 1 mA: 1,275.62 ± 291.74, naïve 3 mA: 848.5 ± 66.83, saline 3 mA: 525.33 ± 101.91, AITC 3 mA: 2,428.8 ± 384.75). The peak amplitude responses in the TGs are shown in Figure 4H. In the comparison of peak amplitude changes, AITC-injected TGs showed significantly increased activity compared to naive and saline-treated TGs (naive 1 mA: 0.10 ± 0.01, saline 1 mA: 0.05 ± 0.01, AITC-treated 1 mA 0.18 ± 0.03, naive 3 mA: 0.15 ± 0.05, saline 3 mA: 0.21 ± 0.04, AITC-treated 3 mA 0.60 ± 0.07). Our data suggest that the AITC group of TGs had higher neuronal activity than the naive or saline group, and the difference was larger at high intensity.

### AITC-Induced Hyperalgesia is Inhibited by the Nav1.7 Channel Blocker ProTxII

**FIGURE 5:**
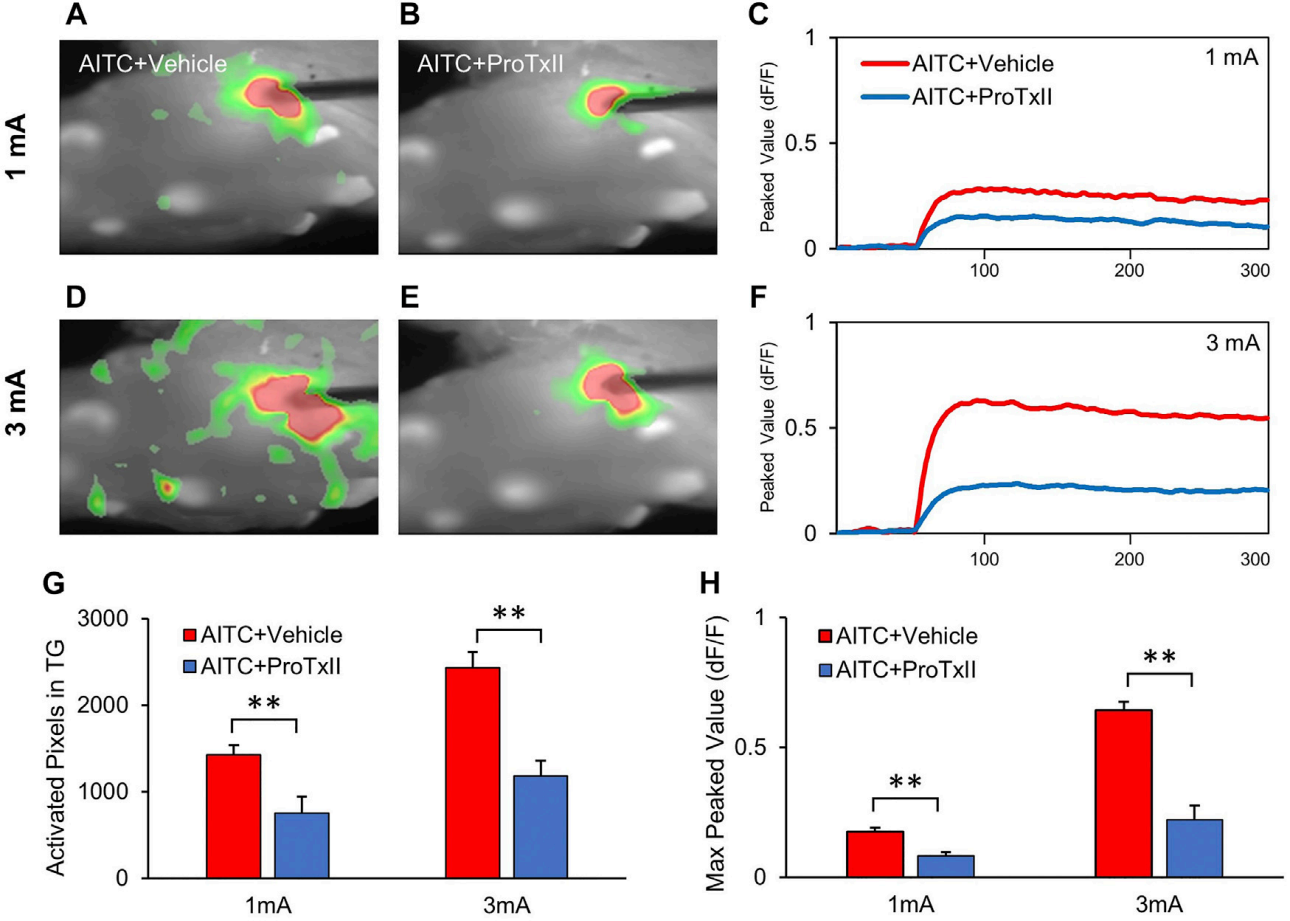
Reduced neuronal activities in TG by application of the ProTxII. **(A,B)** Comparison of optical signals in the inhibition of Nav1.7 activation at 1 mA. **(C,D)** Comparison of optical signal changes after ProTxII at 3 mA. Optical signals showed that the expanded and propagated neuronal activities were reduced after ProTxII. **(E,F)** Temporal peak value changes at 1 and 3 mA. **(G)** Comparison of the activated pixels in the TGs. **(H)** Comparison of the max peak values after ProTxII. Compared to AITC + Vehicle, significantly redeced activated pixcels and peak activity were observed in AITC + ProTxII group (Error bars represent SEM; **p < 0.01).

Optical imaging was performed in the TGs to elucidate the spatiotemporal dynamics of neural activity after ProTxII application. Electrical stimulation-induced neuronal activity of the TGs was observed (Figures 5A,B). Compared to 1 mA stimulation, higher amplitude stimulation 3 mA showed more pronounced and propagated neuronal depolarization in the TGs (Figures 5D,E). The excitatory signal amplitudes were significantly hyperexcited with increasing stimulus intensity, whereas only a slight increase was observed in the 3 mA stimulation with ProTxII (Figure 5C). Excitatory TG responses exhibit sustained neural activation, regardless of stimulus intensity. However, when a stronger stimulus was applied to the TGs, the peak value of the neural excitation increased and maintained (Figure 5F). Figure 5G shows the activation pixels before and after the application of ProTxII (1 mA AITC + vehicle: 1,425.51 ± 109.36, 1 mA AITC + ProTxII: 752.42 ± 185.97; 3 mA AITC + vehicle: 2,429.71 ± 183.68, 3 mA AITC + ProTxII: 1,181.42 ± 173.73). ProTxII inhibited the excitatory neuronal activity by less than 50% of the peaks amplitude of the optical signals compared with AITC only (1 mA AITC: 0.17 ± 0.01, 1 mA AITC + ProTxII: 0.08 ± 0.01; 3 mA AITC: 0.62 ± 0.03, 3 mA AITC + ProTxII: 0.22 ± 0.05) (Figure 5H). These results indicate that the increased neuronal activity generated by Nav1.7 expressions in AITC-treated TGs can be effectively inhibited by ProTxII.

## Discussion

Current research has focused on clarifying the pathogenesis of nociception following peripheral inflammation. In this study, we used AITC to induce an inflammatory response and confirmed the formation of acute pulpitis. Three days after the AITC injection, orofacial pain behaviors related to the inflammatory response were observed. AITC-induced pulpitis pain, as observed in our study, results in increased untypical behaviors such as a nociceptive reaction to the inflammatory response. The results of our study showed the upregulated expression level of Nav1.7 in the TGs during dental pulp inflammation. Moreover, ProTxII, a selective Nav1.7 inhibitor, suppressed the increased neuronal excitability following pulpitis. Therefore, the Nav1.7 channel could modulate nociceptive signal processing in the peripheral nervous system during pulp inflammation.

AITC has been used to examine pulp inflammation in animal models (Kawamura et al., 2010; Narita et al., 2012; Murano et al., 2021). Additionally, explicit abnormal behaviors were observed within 3 days of inflammation. Different analysis techniques can assess the level of pulpal induced-inflammation. The combination of several techniques is useful depending on the objective of the study. The observation of abnormal behavioral reactions, such as flinching, face-washing, chin-rubbing, ear grasps, paw-licking, and facial expressions, in rats has been widely used in several studies as a method of complementary pain assessment (Luccarini et al., 2006; Deuis et al., 2017; Sperry et al., 2018). Although it is difficult to establish an accurate meaning of abnormal behavior, increasing repetitive behavior, such as facial grooming, is considered a reliable symptom of intra-dental pain (Vos et al., 1998; Cha et al., 2020). Furthermore, alterations in voluntary movement and detection of abnormal behaviors following injuries have been recognized as indicators of pain in rats (Chudler and Byers, 2005). Our study demonstrated a significant increase in atypical behaviors such as face-wash strokes, chin rubs, and paw-licks in the AITC-induced pulpitis group, which is consistent with the results of previous study (Cha et al., 2020). As a nociceptive behavior to pulpitis, increased facial grooming appeared more often in the AITC-induced group than in the other groups. Taken together, increasing time spent on abnormal behaviors could be thought to be the result of pulpitis-produced pain.

Inflammatory pain in the oral cavity is induced by the initiation of plastic changes due to central or peripheral sensitization (Sessle, 2000; Tsuboi et al., 2011). Dental pulp inflammation induces central sensitization, which has been shown to increase subcutaneous nociceptive fields and abnormal activity (Chiang et al., 2002; Chiang et al., 2005; Kawamura et al., 2010). Furthermore, this inflammation causes respective receptors to activate intracellular signaling pathways, leading to increased membrane excitability of VGSCs (Amir et al., 2006; Hameed, 2019). Additionally, in the trigeminal nuclei, an increase in VGSCs implicated central sensitization, with higher strength in synaptic links and long-term potentiation (Siqueira et al., 2009). Hence, the role of VGSCs is involved in chronic pain such as inflammatory pain (Rogers et al., 2006; Dib-Hajj et al., 2010). Most subtypes that have been included in this pain pathway are Nav1.7, Nav1.8, and Nav1.9 (Fang et al., 2002; Luo et al., 2010). Among these channel subtypes, the expression of Nav1.7 increased in various chronic pain states (Dib-Hajj et al., 2007; Sun et al., 2019). Consistent with the results of previous studies, our investigation also confirmed that the expression level of Nav1.7 in TGs was upregulated in the pulpitis state. Nav1.7 is abundantly expressed in the DRG and sympathetic ganglion neurons in the peripheral nervous system (Sangameswaran et al., 1997; Toledo-Aral et al., 1997). Inflammation leads to the upregulation of Nav1.7 in DRG neurons, which project to the provoked region (Black et al., 2004; Gould et al., 2004; Strickland et al., 2008). The IHC results have shown that Nav1.7 is highly-expressed in small diameter DRG in TG. Also, our data suggest that enhanced expression and function of Nav1.7 channels in TG neurons resulted in higher excitability and facilitated nociceptive signaling. These results are discussed as leading to an increase in TRPV1 in TG and related satellite cell activation. A large number of satellite cells in TG are known to be activated after tissue inflammation or trigeminal nerve injury (Hanani, 2005). The neuronal activity of TG increased by Nav1.7 causes intracellular ERK phosphorylation, which in turn activates satellite cells around neurons (Black et al., 2012). The activated satellite cells release various molecules, affecting the activity of TG neurons as well as resting satellite cells (Hanani, 2010). This means that pulpitis, which is a result of the inflammatory response of molar 1, consistently induces changes in Nav1.7 that are sufficient to indicate pain in the TG.

In this study, the results of immunostaining and WB data proved that the inflammatory response induced by molar 1 caused not only acute pain but also a chronic increase of Nav1.7 expressions in the TG. The deletion of Nav1.7 (Nav1.7 knockout) in all sensory neurons caused an additional deprivation of noxious thermal sensation (Minett et al., 2012; Kanellopoulos and Matsuyama, 2016). These studies showed the significance of Nav1.7 in pain-related sensations and suggested it as an essential drug target for pain therapies. In the present study, 3 days after pulp inflammation formation, notably increased neuronal activity was detected in the TGs in the AITC group compared to that in the naive and saline-injected groups. Since the AITC group exhibited inflammatory response, pain-related behaviors and increased Nav1.7 expression, it could be concluded that prior results are correlated with the increase in the neuronal excitability of the TG. It has been established that the inflammatory response is sharply reduced when Nav1.7 is knocked out in animal sensory neurons, which are known to be important pathways in the development of inflammatory pain (Nassar et al., 2004; Dib-Hajj et al., 2010). These previous findings are in accordance with our result that the upregulation of neuronal activation in the TGs is related to the increased expression of Nav1.7.

ProTxII, which is extracted from the tarantula, has been proved as a blocker of Nav1.7 (Middleton et al., 2002; Smith et al., 2007). Previous studies have indicated that ProTxII affects inflammation-induced hyperalgesia (Schmalhofer et al., 2008; Emery et al., 2016). In this study, we investigated whether ProTxII inhibits neuronal hyperexcitability by using optical imaging of the TGs in an AITC-induced pulpitis model. We found that ProTxII suppressed excitatory neuronal activity. Moreover, we confirmed that the increased propagation of neuronal activity induced by Nav1.7 expressions in the TGs could be effectively blocked by ProTxII.

Recent animal and clinical studies strongly support that Nav1.7 plays an important role in the pathophysiology of dental disease, such as burning mouth syndrome (Beneng et al., 2010), inflamed TMJ (Bi et al., 2017), trigeminal neuralgia (Zakrzewska et al., 2017), and pulpitis (Aubeux et al., 2021). The high levels of Nav1.7 expressions may be due to pulpitis-induced inflammatory factors and overexpressed interaction of satellite cell-neuron and/or neuron-neuron. The results of our study indicated the inhibition of Nav1.7 channel activities possible clinical application target of pulpitis. Important roles of Nav1.7 in inflammatory pain mechanisms can be a novel target for neuronal excitability, which is related to various pathological circumstances. Although inhibition of Nav1.7 is effective for pain relief, Nav1.7-based drug development for pulpitis-induced pain regulation has not yet been studied. Further research is necessary for the exploitation of Nav1.7 blockers, an upcoming prospective therapeutic strategy for inflammatory pain. Understanding the specific roles of Nav1.7 may contribute to fulfilling the demands for more accurate and effective medicaments for patients with inflammatory pain.

## Data Availability

The original contributions presented in the study are included in the article/Supplementary Material, further inquiries can be directed to the corresponding authors.
